# Managing the Risks of Extreme Weather: IPCC Special Report

**DOI:** 10.1289/ehp.120-a58

**Published:** 2012-02-01

**Authors:** Catherine M. Cooney

**Affiliations:** Catherine M. Cooney is a science writer living in Washington, DC, who has contributed to *Environmental Science & Technology* and other publications.

The number of hot days and nights very likely has increased globally in recent years, according to a special report^1^ focused solely on extreme weather events from the Intergovernmental Panel on Climate Change (IPCC),^2^ while the number of cold days has decreased. The future looks similar, the IPCC panel says: If countries continue to increase emissions of carbon dioxide (CO_2_)—the greenhouse gas produced by human activities in the greatest quantities—deadly heat waves and heavy precipitation events will occur more often. Devastating tropical cyclones, on the other hand, are likely to remain the same or even decrease.

A summary report for policy makers was released 18 November 2011 in advance of the February 2012 publication of the full IPCC Special Report *Managing the Risks of Extreme Events and Disasters to Advance Climate Change Adaptation* (SREX). Several aspects of SREX are designed to inform governments and other decision makers struggling to develop climate-change adaptation plans. The report offers adaptation measures that planners can implement to protect human health during extreme weather events. These include “low-regrets” activities that provide benefits now and under a variety of future scenarios, such as installing systems that warn people of impending disasters and improving systems for health surveillance, drinking water, and drainage.

This publication represents the first time that IPCC working groups I (which focuses on the physical science basis of climate change) and II (which focuses on impacts, adaptation, and vulnerability) have collaborated on a report, says SREX coordinating lead author Sonia I. Seneviratne, an assistant professor at the Institute for Atmospheric and Climate Science, ETH Zürich. It also includes several lead authors from the disaster risk management community. “I think the report allows a better integration of information all the way from the physical projections of climate extremes to disaster management and climate adaptation options. This should make it particularly valuable for decision makers,” Seneviratne says.

The analysis concludes that extreme weather events will particularly affect sectors closely tied to climate: water, agriculture, food security, forestry, health, and tourism. The severity of human health impacts from climate extremes will reflect how prepared or how vulnerable a community is. For example, people living in areas with rapid and unplanned urbanization, environmental degradation, and poverty are more vulnerable to the hazards of extreme climate events than those living in better-planned, better-protected, and higher-income communities. After a disaster, the summary notes, planners should focus on reconstruction that improves a community’s resistance to weather- and climate-related disasters rather than recreating or even worsening existing vulnerabilities.

The report helps untangle some of the confusion nonscientists feel when reading news reports about blockbuster blizzards at the same time the Earth is supposedly warming.^3^ Gerald A. Meehl, senior scientist at the National Center for Atmospheric Research (NCAR) and a lead author on the near-term climate change chapter for the forthcoming IPCC Fifth Assessment Report (due by 2014), explains that the very nature of global warming exacerbates extreme weather events of all kinds—not just heat-related events.

“We know that CO_2_ traps heat in the atmosphere,” Meehl says. “That causes things to warm up, and warmer air holds more moisture, which means there is more moisture available as a source for precipitation in storms.” Precipitation intensity increases, he says, even though the overall number of storms may not increase. Even in a much warmer climate, Meehl adds, there will still be record cold temperatures and snow storms. However, as the atmosphere continues to warm, “extreme cold will occur less frequently than extreme heat,”^4^ he says.

At least one scientist thinks the SREX underestimates the extent to which human activity affects climate. Kevin Trenberth, distinguished senior scientist in the Climate Analysis Section at the NCAR, says the report “inherently assumes a null hypothesis of no human influence. In reality, many studies have shown otherwise.”

Combined with short-term data sets that often contain variabilities and the fact that many models don’t accurately simulate certain extremes such as tropical storms and monsoons, the message would appear to be there is no human influence, according to Trenberth. “The result of the null hypothesis is that the errors from imperfect models and data fall on the side of saying there is no human influence when there really is,” he says. “This is a fundamental issue with the science community as well as public perceptions. . . . The result has been the appearance of overwhelming uncertainties and paralysis of action.”

**Figure:**
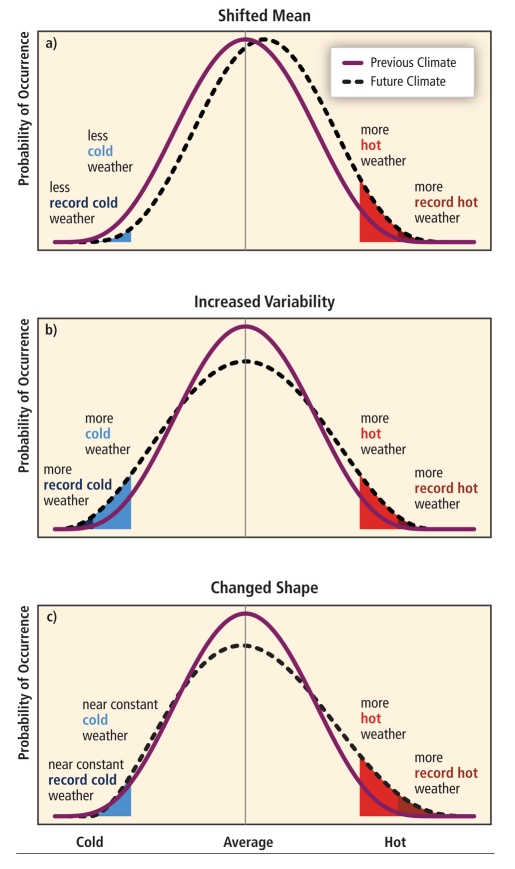
Changes in the mean, variance, or shape of weather probability distributions—or some combination of those three—could mean a change in the number and severity of extreme weather events. (Source: IPCC^1^)

## References

[1] IPCC (2011). Summary for Policymakers. In: Intergovernmental Panel on Climate Change Special Report on Managing the Risks of Extreme Events and Disasters to Advance Climate Change Adaptation (Field CB, et al., eds). http://ipcc-wg2.gov/SREX/.

[2] The IPCC is the scientific body tasked with evaluating the risk of climate change caused by human activity, the scientific basis for climate change, and the mitigation options.

[3] NASA Earth Observatory [website]. Greenbelt, MD:NASA Earth Observatory, EOS Project Science Office, National Aeronautics and Space Administration (acquired 7 Feb 2010). Available: http://earthobservatory.nasa.gov/IOTD/view.php?id=42568 [accessed 2 Dec 2011].

[4] Meehl GA (2009). Relative increase of record high maximum temperatures compared to record low minimum temperatures in the U.S.. Geophys Res Lett.

